# *Stilbocrea walteri* sp. nov., an unusual species of Bionectriaceae

**DOI:** 10.1007/s11557-018-1427-0

**Published:** 2018-08-04

**Authors:** Hermann Voglmayr, Walter M. Jaklitsch

**Affiliations:** 1 Division of Systematic and Evolutionary Botany, Department of Botany and Biodiversity Research, University of Vienna, Rennweg 14, 1030 Wien, Austria; 2 Institute of Forest Entomology, Forest Pathology and Forest Protection, Department of Forest and Soil Sciences, BOKU-University of Natural Resources and Life Sciences, Franz Schwackhöfer Haus, Peter-Jordan-Straße 82/I, 1190 Wien, Austria

**Keywords:** Ascomycota, Hypocreales, *Nectria*, Phylogenetic analysis, Sordariomycetes, Taxonomy

## Abstract

The new species *Stilbocrea walteri* is described and illustrated from *Quercus ilex* collected in Portugal. Phylogenetic analyses of LSU rDNA, *rpb1*, *rpb2* and *tef1* sequence matrices place *S. walteri* in the Bionectriaceae, Hypocreales, within a clade of specimens morphologically identified as *Stilbocrea macrostoma*, the generic type of *Stilbocrea*. *Stilbocrea walteri* differs from *S. macrostoma* in dark olive green to blackish ascomata basally immersed in a stroma, KOH+ and LA+ ascomata and the lack of a stilbella-like asexual morph on natural substrate and pure culture. A simple phialidic asexual morph is formed in pure culture. To enable a morphological comparison, *Stilbocrea macrostoma* is illustrated.

## Introduction

During a collecting trip to Portugal, a black stromatic pyrenomycete was collected on dead corticated branches of *Quercus ilex*. Microscopic analyses revealed a nectriaceous fungus, which could not be identified to genus or species, and also the familial affiliation remained unclear. The partial immersion of ascomata in a well-developed stroma and reddening of the ascomatal walls in KOH pointed towards Nectriaceae, but molecular phylogenetic analysis based on LSU rDNA, *rpb1*, *rpb2* and *tef1* sequences revealed a placement within Bionectriaceae. Based on this evidence, a new species of *Stilbocrea* is described.

## Materials and methods

### Culture preparation, isolates and specimens

Cultures were prepared from ascospores and maintained as described previously (Jaklitsch 2009). Germinating ascospores were placed on CMD (CMA: Sigma, St Louis, Missouri; supplemented with 2% (*w*/*v*) D(+)-glucose-monohydrate) or 2% malt extract agar (MEA; 2% *w*/*v* malt extract, 2% *w*/*v* agar-agar; Merck, Darmstadt, Germany). The plates were sealed with laboratory film and incubated at room temperature. Cultures used for the study of the asexual morph were grown on 2% MEA or CMD at room temperature (22 ± 3 °C) under alternating 12 h daylight and 12 h darkness. The ex-type culture was deposited at the Westerdijk Fungal Biodiversity Centre (CBS-KNAW), Utrecht, The Netherlands, and specimens in the Fungarium of the Institute of Botany, University of Vienna (WU). The following specimens of *Stilbocrea macrostoma* were sequenced for the phylogenetic analyses and/or used for morphological illustration and comparison but are not described in detail here: Panama, Parque Nacional Altos de Campana, on dead branch of an unidentified tree, 5 May 2012, E. Esquivel (WU 32032); culture SM, prepared and maintained on PDA (Merck). Sri Lanka, Nuwara Eliya, Hakgala Sanctuary Botanical Gardens, 12 Feb. 1984, I. Krisai-Greilhuber IK 2346 (WU 26101).

### Morphological observations

Microscopic preparations were mounted in water, 3% potassium hydroxide (KOH) or lactic acid (LA). Stereomicroscopy illustrations and measurements were done with a Keyence VHX-6000 system. Light microscopy was performed with Nomarski differential interference contrast (DIC) using the Zeiss Axio Imager.A1 compound microscope, and images and data were gathered using the Zeiss Axiocam 506 colour digital camera and measured by using the Zeiss ZEN Blue Edition software. Measurements are reported as maxima and minima in parentheses and the mean plus and minus the standard deviation of a number of measurements given in parentheses.

### DNA extraction, PCR and sequencing

Growth of liquid culture and extraction of genomic DNA was done according to Voglmayr and Jaklitsch (2011), using the DNeasy Plant Mini Kit (QIAgen GmbH, Hilden, Germany). To confirm the identity of the culture, DNA was also extracted from stromata following the protocol of Voglmayr and Jaklitsch (2011) for herbarium specimens, but using the DNeasy Plant Mini Kit. The complete ITS region and D1 and D2 domains of 28S rDNA region (ITS-LSU) were amplified with primers V9G (de Hoog and Gerrits van den Ende 1998) and LR5 (Vilgalys and Hester 1990), a ca. 750 bp fragment of the RNA polymerase II subunit 1 (*rpb1*) gene with primers RPB1-Ac (Schoch et al. 2012) and RPB1Cr (Sung et al. 2007b), a ca. 1.1 kb fragment of the RNA polymerase II subunit 2 (*rpb2*) gene with primers fRPB2-5F and fRPB2-7cR (Liu et al. 1999) or dRPB2-5f and dRPB2-7r (Voglmayr et al. 2016) and a ca. 1.4 kb fragment of the translation elongation factor 1-α (*tef1*) gene with primers EF1-728F (Carbone and Kohn 1999) and EF1-2218R (Rehner and Buckley 2005). From stromatal DNA, only the ITS-LSU was amplified and sequenced. PCR was performed with a Taq polymerase, with annealing temperatures of 55 °C for ITS-LSU, *tef1* and *rpb2* (primer pair fRPB2-5F, fRPB2-7cR) and 51 °C for *rpb1* and *rpb2* (primer pair dRPB2-5f, dRPB2-7r). PCR products were purified using an enzymatic PCR cleanup (Werle et al. 1994) as described in Voglmayr and Jaklitsch (2008). DNA was cycle-sequenced using the ABI PRISM Big Dye Terminator Cycle Sequencing Ready Reaction Kit v. 3.1 (Applied Biosystems, Warrington) and the PCR primers; in addition, primers ITS4 (White et al. 1990), LR3 (Vilgalys and Hester 1990) and LR2R-A (Voglmayr et al. 2012) were used for the ITS-LSU region. Sequencing was performed on an automated DNA sequencer (ABI 3730xl Genetic Analyser, Applied Biosystems).

### Phylogenetic analyses

**Fig. 1:**
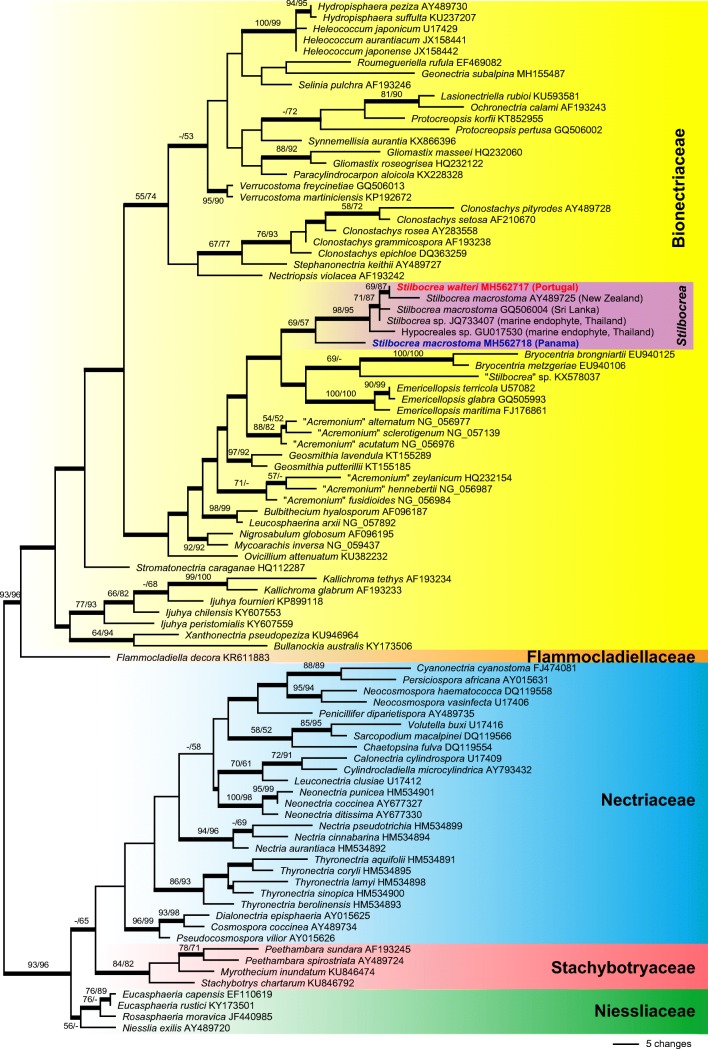
Phylogram obtained by PAUP from an analysis of the LSU matrix of selected Hypocreales, showing one of 24 most parsimonious trees 1202 steps long. *Stilbocrea walteri* is revealed as a species of the Bionectriaceae. GenBank accession numbers of sequences are given following the taxon names. The country of origin is provided for accessions within the *Stilbocrea* clade. Isolates in bold were sequenced during the present study; thickened internal branches are present in the strict consensus of all 24 MP trees. MP and ML bootstrap support above 50% are given at first and second position, respectively, above or below the branches

**Fig. 2:**
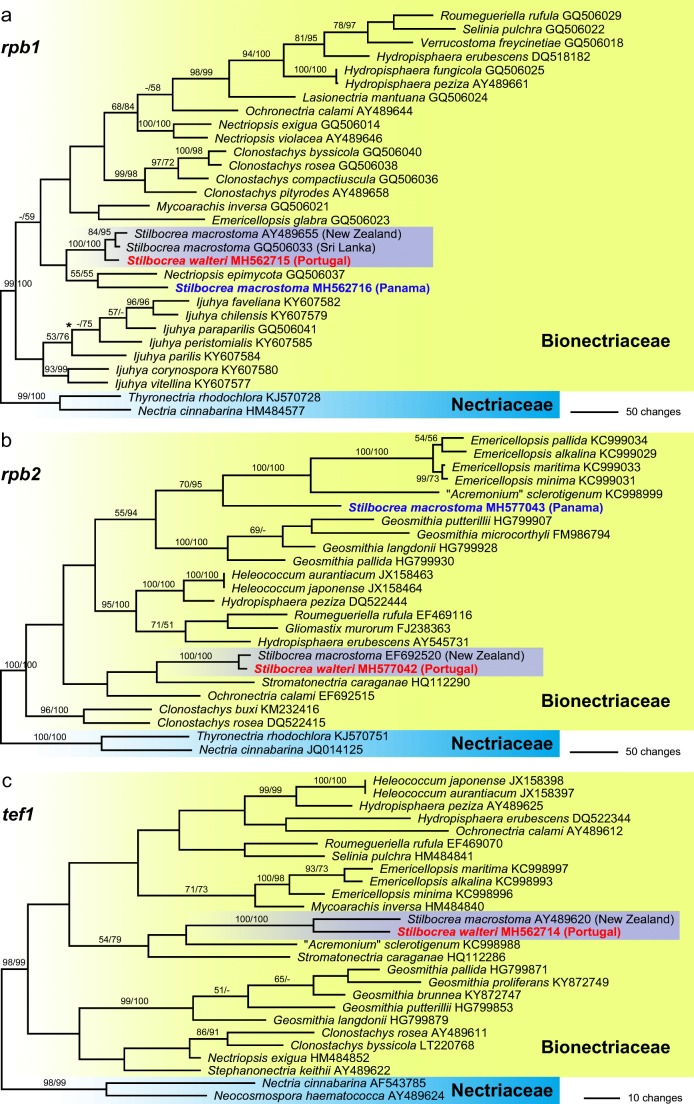
Phylograms revealed by PAUP from MP analyses of the *rpb1* (**a**), *rpb2* (**b**) and *tef1* (**c**) matrices, showing the phylogenetic position of *Stilbocrea walteri* within Bionectriaceae. **a** One of two MP trees 2320 steps long; asterisk (*) denoting node that collapsed in the strict consensus of the two MP trees. **b** Single MP tree 2597 steps long. **c** Single MP tree 957 steps long. GenBank accession numbers of sequences are given following the taxon names; isolates in bold were sequenced during the present study. MP and ML bootstrap support above 50% are given at first and second position, respectively, above or below the branches

As the LSU rDNA is the most representative marker available for many genera of Bionectriaceae, an extended LSU matrix was produced for phylogenetic analyses. For this, the sequence matrix of Jaklitsch and Voglmayr (2011a) was supplemented with selected sequences from Summerbell et al. (2011) and a few additional GenBank sequences. Only few *rpb1*, *rpb2* and *tef1* sequences of Bionectriaceae were available from GenBank to phylogenetically place *Stilbocrea*. For the same reason, ITS rDNA was not phylogenetically analysed. The GenBank accession numbers of sequences downloaded for phylogenetic analyses are given in Table 1 and in the phylogenetic trees (Figs. 1 and 2), following the taxon names. Generic classification of the Nectriaceae follows Lombard et al. (2015), of Stachybotryaceae Lombard et al. (2016) and of Bionectriaceae the taxonomy implemented in NCBI GenBank, with a few additions of recently published new genera.

**Table 1:** List of taxa and GenBank accessions used in the current phylogenetic study. The references are according to the NCBI Nucleotide database. Sequences in bold were generated during the present study

Taxon	LSU	*rpb1*	*rpb2*	*tef1*	References
“*Acremonium*” *acutatum*	NG_056976				Summerbell et al. (2011)
“*Acremonium*” *alternatum*	NG_056977				Summerbell et al. (2011)
“*Acremonium*” *fusidioides*	NG_056984				Summerbell et al. (2011)
“*Acremonium*” *hennebertii*	NG_056987				Summerbell et al. (2011)
“*Acremonium*” *sclerotigenum*	NG_057139		KC998999	KC998988	Hijikawa et al. (2017), Grum-Grzhimaylo et al. (2013b)
“*Acremonium*” *zeylanicum*	HQ232154				Summerbell et al. (2011)
*Bryocentria brongniartii*	EU940125				Stenroos et al. (2010)
*Bryocentria metzgeriae*	EU940106				Stenroos et al. (2010)
*Bulbithecium hyalosporum*	AF096187				Suh and Blackwell (1999)
*Bullanockia australis*	KY173506				Crous et al. (2016a)
*Calonectria cylindrospora*	U17409				Rehner and Samuels (1995)
*Chaetopsina fulva*	DQ119554				Zhang and Zhuang (unpubl.)
*Clonostachys buxi*			KM232416		Lombard et al. (2015)
*Clonostachys byssicola*		GQ506040		LT220768	Hirooka et al. (2010), Sharma and Marques (unpubl.)
*Clonostachys compactiuscula*		GQ506036			Hirooka et al. (2010)
*Clonostachys epichloe*	DQ363259				Kirschner (unpubl.)
*Clonostachys grammicospora*	AF193238				Rossman et al. (2001)
*Clonostachys pityrodes*	AY489728	AY489658			Castlebury et al. (2004)
*Clonostachys rosea*	AY283558	GQ506038	DQ522415	AY489611	Seifert et al. (2003), Hirooka et al. (2010), Spatafora et al. (2007), Castlebury et al. (2004)
*Clonostachys setosa*	AF210670				Schroers (2001)
*Cosmospora coccinea*	AY489734				Castlebury et al. (2004)
*Cyanonectria cyanostoma*	FJ474081				Samuels et al. (unpubl.)
*Cylindrocladiella microcylindrica*	AY793432				Crous et al. (2005)
*Dialonectria episphaeria*	AY015625				Zhang and Blackwell (2002)
*Emericellopsis alkalina*			KC999029	KC998993	Grum-Grzhimaylo et al. (2013)
*Emericellopsis glabra*	GQ505993	GQ506023			Hirooka et al. (2010)
*Emericellopsis maritima*	FJ176861		KC999033	KC998997	Grum-Grzhimaylo et al. (2013b)
*Emericellopsis minima*			KC999031	KC998996	Grum-Grzhimaylo et al. (2013b)
*Emericellopsis pallida*			KC999034		Grum-Grzhimaylo et al. (2013b)
*Emericellopsis terricola*	U57082				Glenn and Bacon (unpubl.)
*Eucasphaeria capensis*	EF110619				Crous et al. (2007)
*Eucasphaeria rustici*	KY173501				Crous et al. (2016a)
*Flammocladiella decora*	NG_058175				Crous et al. (2015a)
*Geonectria subalpina*	MH155487				Lechat et al. (2018)
*Geosmithia brunnea*				KY872747	Huang et al. (unpubl.)
*Geosmithia langdonii*			HG799928	HG799879	Kolarik et al. (unpubl.)
*Geosmithia lavendula*	KT155289				Stielow et al. (unpubl.)
*Geosmithia microcorthyli*			FM986794		Kolarik and Kirkendall (2010)
*Geosmithia pallida*			HG799930	HG799871	Kolarik et al. (unpubl.)
*Geosmithia proliferans*				KY872749	Huang et al. (unpubl.)
*Geosmithia putterillii*	KT155185		HG799907	HG799853	Stielow et al. (unpubl.), Kolarik et al. (unpubl.)
*Gliomastix masseei*	HQ232060				Summerbell et al. (2011)
*Gliomastix murorum*			FJ238363		Schoch et al. (unpubl.)
*Gliomastix roseogrisea*	HQ232122				Summerbell et al. (2011)
*Heleococcum aurantiacum*	JX158463		JX158463	JX158397	Grum-Grzhimaylo et al.(2013a)
*Heleococcum japonense*	JX158442		JX158464	JX158398	Grum-Grzhimaylo et al.(2013a)
*Heleococcum japonicum*	U17429				Rehner and Samuels (1995)
*Hydropisphaera erubescens*		DQ518182	AY545731	DQ522344	James et al. (unpubl.), AFTOL (unpubl.), Spatafora et al. (2007)
*Hydropisphaera fungicola*		GQ506025			Hirooka et al. (2010)
*Hydropisphaera peziza*	AY489730	AY489661	DQ522444	AY489625	Castlebury et al. (2004), Spatafora et al. (2007)
*Hydropisphaera suffulta*	KU237207				Lechat (unpubl.)
Hypocreales sp.	GU017530				Sakayaroj et al. (2010)
*Ijuhya chilensis*	KY607553	KY607579			Ashrafi et al. (2017)
*Ijuhya corynospora*		KY607580			Ashrafi et al. (2017)
*Ijuhya faveliana*		KY607582			Ashrafi et al. (2017)
*Ijuhya fournieri*	KP899118				Lechat et al. (2015)
*Ijuhya paraparilis*		GQ506041			Hirooka et al. (2010)
*Ijuhya parilis*		KY607584			Ashrafi et al. (2017)
*Ijuhya peristomialis*	KY607559	KY607585			Ashrafi et al. (2017)
*Ijuhya vitellina*		KY607577			Ashrafi et al. (2017)
*Kallichroma glabrum*	AF193233				Rossman et al. (2001)
*Kallichroma tethys*	AF193234				Rossman et al. (2001)
*Lasionectria mantuana*		GQ506024			Rossman et al. (2001)
*Lasionectriella rubioi*	KU593581				Lechat and Fournier (2016)
*Leuconectria clusiae*	U17412				Rehner and Samuels (1995)
*Leucosphaerina arxii*	NG_057892				Summerbell et al. (2011)
*Mycoarachis inversa*	NG_059437	GQ506021		HM484840	Hirooka et al. (2010), Chaverri et al. (2011)
*Myrothecium inundatum*	KU846474				Lombard et al. (2016)
*Nectria aurantiaca*	HM534892				Jaklitsch and Voglmayr (2011b)
*Nectria cinnabarina*	HM534894	HM484577	JQ014125	AF543785	Jaklitsch and Voglmayr (2011b), Hirooka et al. (2011), Schoch et al. (2012), Currie et al. (2003)
*Nectria pseudotrichia*	HM534899				Jaklitsch and Voglmayr (2011b)
*Nectriopsis epimycota*		GQ506037			Hirooka et al. (2010)
*Nectriopsis exigua*		GQ506014		HM484852	Hirooka et al. (2010), Chaverri et al. (2011)
*Nectriopsis violacea*	AF193242	AY489646			Rossman et al. (2001), Castlebury et al. (2004)
*Neocosmospora haematococca*	DQ119558			AY489624	Zhang and Zhuang (unpubl.), Castlebury et al. (2004)
*Neocosmospora vasinfecta*	U17406				Rehner and Samuels (1995)
*Neonectria coccinea*	AY677327				Halleen et al. (2004)
*Neonectria ditissima*	AY677330				Halleen et al. (2004)
*Neonectria punicea*	HM534901				Jaklitsch and Voglmayr (2011b)
*Niesslia exilis*	AY489720				Castlebury et al. (2004)
*Nigrosabulum globosum*	AF096195				Suh and Blackwell (1999)
*Ochronectria calami*	AF193243	AY489644	EF692515	AY489612	Rossman et al. (2001), Castlebury et al. (2004), Sung et al. (2008)
*Ovicillium attenuatum*	KU382232				Zare and Gams (2016)
*Paracylindrocarpon aloicola*	KX228328				Crous et al. (2016b)
*Peethambara spirostriata*	AY489724				Castlebury et al. (2004)
*Peethambara sundara*	AF193245				Rossman et al. (2001)
*Penicillifer diparietispora*	AY489735				Castlebury et al. (2004)
*Persiciospora africana*	AY015631				Zhang and Blackwell (2002)
*Protocreopsis korfii*	KT852955				Lechat and Fournier (2015)
*Protocreopsis pertusa*	GQ506002				Hirooka et al. (2010)
*Pseudocosmospora vilior*	AY015626				Zhang and Blackwell (2002)
*Rosasphaeria moravica*	JF440985				Jaklitsch and Voglmayr (2012)
*Roumegueriella rufula*	EF469082	GQ506029	EF469116	EF469070	Sung et al. (2007a), Hirooka et al. (2010)
*Sarcopodium macalpinei*	DQ119566				Zhang and Zhuang (unpubl.)
*Selinia pulchra*	AF193246	GQ506022		HM484841	Rossman et al. (2001), Hirooka et al. (2010), Chaverri et al. (2011)
*Stachybotrys chartarum*	KU846792				Lombard et al. (2016)
*Stephanonectria keithii*	AY489727			AY489622	Castlebury et al. (2004)
*Stilbocrea macrostoma*	AY489725, GQ506004, **MH562716**	GQ506033, AY489655, **MH562716**	EF692520, **MH577043**	AY489620	Hirooka et al. (2010), Castlebury et al. (2004), Sung et al. (2008), this study
*Stilbocrea* sp.	JQ733407				Supaphon et al. (2017)
“*Stilbocrea*” sp.	KX578037				Lechat (unpubl.)
*Stilbocrea walteri*	**MH562717**	**MH562715**	**MH577042**	**MH562714**	this study
*Stromatonectria caraganae*	HQ112287		HQ112290	HQ112286	Jaklitsch and Voglmayr (2011a)
*Synnemellisia aurantia*	KX866396				Lisboa et al. (unpubl.)
*Thyronectria aquifolii*	HM534891				Jaklitsch and Voglmayr (2011b)
*Thyronectria berolinensis*	HM534893				Jaklitsch and Voglmayr (2011b)
*Thyronectria coryli*	HM534895				Jaklitsch and Voglmayr (2011b)
*Thyronectria lamyi*	HM534898				Jaklitsch and Voglmayr (2011b)
*Thyronectria rhodochlora*		KJ570728	KJ570751		Jaklitsch and Voglmayr (2014)
*Thyronectria sinopica*	HM534900				Jaklitsch and Voglmayr (2011b)
*Verrucostoma freycinetiae*	GQ506013	GQ506018			Hirooka et al. (2010)
*Verrucostoma martiniciensis*	KP192672				Crous et al. (2015b)
*Volutella buxi*	U17416				Rehner and Samuels (1995)
*Xanthonectria pseudopeziza*	KU946964				Lechat et al. (2016)

The downloaded GenBank sequences were aligned with the sequences generated in our study with the server version of MAFFT (www.ebi.ac.uk/Tools/mafft) using the default settings and checked and refined with BioEdit v. 7.0.9.0 (Hall 1999). The four matrices were analysed separately. The final matrices used for phylogenetic analyses contained 863, 750, 1072 and 951 alignment characters for the LSU, *rpb1*, *rpb2* and *tef1*, respectively.

Maximum parsimony (MP) analyses were performed with PAUP v. 4.0a161 (Swofford 2002), using 1000 replicates of heuristic search with random addition of sequences and subsequent TBR branch swapping (MULTREES option in effect, steepest descent option not in effect). All molecular characters were unordered and given equal weight; analyses were performed with gaps treated as missing data; the COLLAPSE command was set to MAXBRLEN. Bootstrap analysis with 1000 replicates was performed in the same way, but using 5 rounds of random sequence addition and subsequent TBR branch swapping during each bootstrap replicate, with the COLLAPSE command set to MINBRLEN; in addition, each replicate was limited to 1 million rearrangements in the LSU analyses. All molecular characters were unordered and given equal weight; analyses were performed with gaps treated as missing data; the COLLAPSE command was set to minbrlen.

Maximum likelihood (ML) analyses were performed with RAxML (Stamatakis 2006) as implemented in raxmlGUI 1.3 (Silvestro and Michalak 2012) using the ML + rapid bootstrap setting and the GTRGAMMA substitution model with 1000 bootstrap replicates.

Bootstrap support below 70% was considered low, between 70 and 90% moderate and above 90% high.

## Results

### Sequencing and molecular phylogeny

The ITS-LSU sequences obtained from the culture and the stromata of the newly described fungus were identical. Sequence similarity of the ITS of the newly described fungus and the newly sequenced *Stilbocrea macrostoma* accession from Panama (SM) was 83.5% (71 nucleotide substitutions and 14 gaps).

Of the 866 nucleotide characters included in the LSU analyses, 163 were parsimony informative. Maximum parsimony analyses revealed 24 MP trees 1202 steps long, one of which is shown as Fig. 1. The MP trees differed mainly in the deeper nodes of Nectriaceae (Fig. 1); in some of the MP trees, Stachybotryaceae were embedded within the Nectriaceae (not shown). In the phylogenetic analyses, the Stachybotryaceae were moderately supported, while the clade comprising Bionectriaceae plus Flammocladiellaceae received high support. The Flammocladiellaceae were revealed as sister group to Bionectriaceae in the MP analyses; however, the latter did not receive significant bootstrap support (Fig. 1). Within Bionectriaceae, backbone support of deeper nodes was mostly low or absent. The GenBank accessions of *Stilbocrea* included in our LSU analyses did not form a monophylum as the unpublished accession KX578037 from Spain labelled *Stilbocrea* sp. was placed outside the *Stilbocrea* clade. The three accessions of *Stilbocrea macrostoma*, the fungus from Portugal and two GenBank accessions of endophyte isolates from tropical marine seagrasses (JQ733407; GU017530) formed a monophylum with low support (Fig. 1). However, the various accessions of *Stilbocrea macrostoma* did not form a monophylum, as the newly sequenced *S. macrostoma* specimen from Panama was in a basal position to a highly supported subclade containing the new *Stilbocrea* species from Portugal, the GenBank accessions of *S. macrostoma* from New Zealand and Sri Lanka and the two endophyte isolates.

Of the 750 nucleotide characters included in the *rpb1* analyses, 367 were parsimony informative. Maximum parsimony analyses revealed two MP trees 2320 steps long, one of which is shown as Fig. 2a. The two MP trees were identical except for an interchanged position of *Ijuhya peristomialis* and *Ijuhya parilis* (not shown). Of the 1072 nucleotide characters included in the *rpb2* analyses, 533 were parsimony informative. Maximum parsimony analyses revealed a single MP tree 2597 steps long which is shown as Fig. 2b. Of the 951 nucleotide characters included in the *tef1* analyses, 231 were parsimony informative. Maximum parsimony analyses revealed a single MP tree 957 steps long which is shown as Fig. 2c.

In the analyses of the protein-coding genes (*rpb1*, *rpb2*, *tef1*), many of the deeper nodes within Bionectriaceae received no or low support (Fig. 2a–c), and only limited comparisons are possible between these trees due to a different taxon selection. However, the new fungus from Portugal and the GenBank accessions of *Stilbocrea macrostoma* from New Zealand (all three markers available) and Sri Lanka (only *rpb1* available) consistently formed a clade with maximum support (Fig. 2a–c), while the newly sequenced Panamese accession of *Stilbocrea macrostoma* was not contained in this clade (Fig. 2a, b). Remarkably, in the *rpb1* tree (Fig. 2a), the fungus from Portugal was placed in a sister group position to the GenBank accessions of *Stilbocrea macrostoma* from New Zealand and Sri Lanka with medium (84%; MP) to high (95%; ML) support.

Considering morphological and molecular data, the specimen from Portugal is described as a new species.

### Taxonomy

**Fig. 3:**
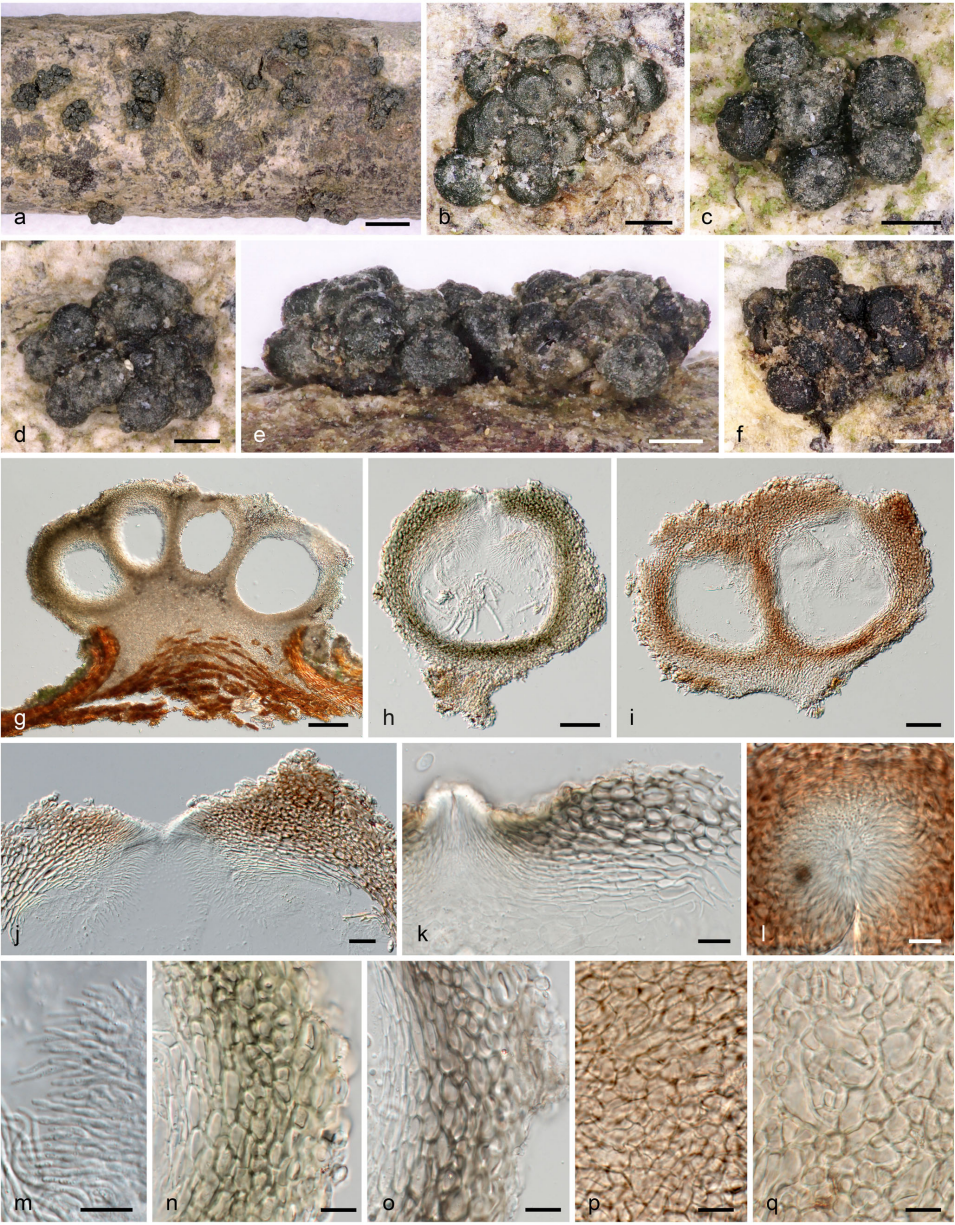
*Stilbocrea walteri*, sexual morph (WU 39972). **a**–**f** Stromata/ascomata. **g**–**i** Stromata in vertical section. **j**, **k** Ostiolar region in vertical section. **l** Ostiole in face view. **m** Periphyses. **n**, **o** Peridium in vertical section. **p** Peridium in face view. **q** Stroma tissue in vertical section (**f**, **i**, **j**, **l**, **m**, **p** in 3% KOH; **g**, **h**, **n**, **q** in water; **k**, **o** in LA). *Scale bars***a** 1 mm; **b–f** 200 μm; **g** 100 μm; **h**, **i** 50 μm; **j** 20 μm; **k**–**q** 10 μm

**Fig. 4:**
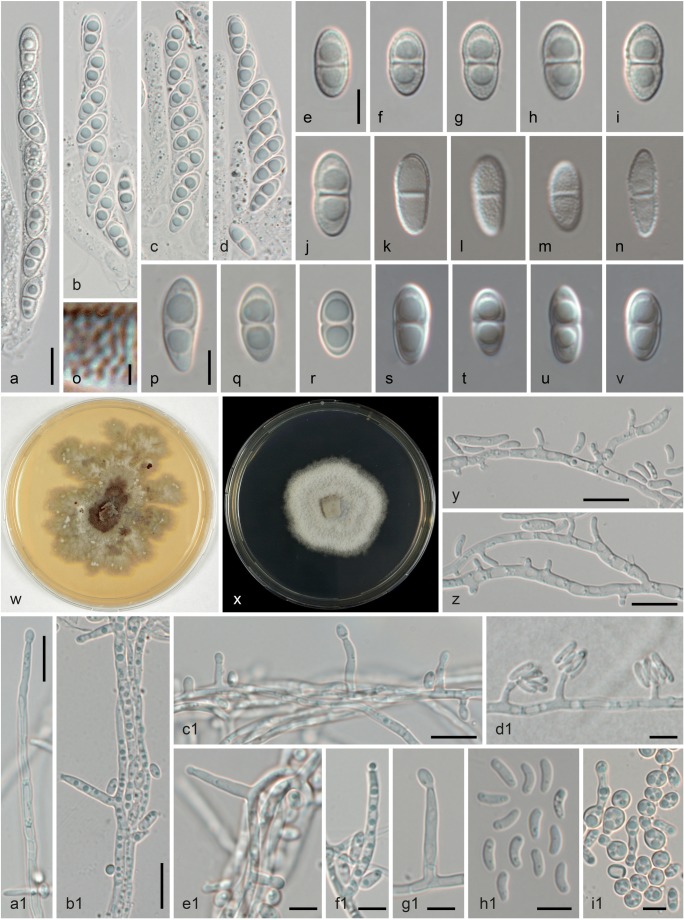
*Stilbocrea walteri*, sexual morph (WU 39972), cultures and asexual morph (NQI = CBS 144627). a–d Asci with ascospores (b–d in 3% KOH). e–n, p–v Ascospores (e–j vital, k–n in LA; p–v in 3% KOH; note verruculose and smooth ascospore walls in water/LA and KOH, respectively). o Detail of verruculose ascospore wall (in LA). w, x Cultures (w MEA, 31 d; x CMD, 20 d). y–g1 Conidiophores, pegs and phialides (y, z, d1 CMD, 4 days; a1–g1 CMD, 20 days). h1 Conidia (CMD, 4 days); i1 Blastoconidia (CMD, 20 days). (all in water except where noted). *Scale bars***a–d**, **y–c1** 10 μm; **e–n**, **p–v**, **d1–i1** 5 μm; **o** 1 μm

***Stilbocrea walteri*** Voglmayr & Jaklitsch, sp. nov. Figs. 3 and 4.

MycoBank MB 826919.

Etymology: in honour of Walter Gams.

Stromata when dry (460–)680–1100(–1600) μm diam (*n* = 50), (260–)300–430(–520) μm high (*n* = 30), scattered, less commonly in groups of 2–3, erumpent from bark, pulvinate; round, elliptical or irregular in outline. Stromata at the base compact, white in section. Perithecia (2–)5–15(–20) per stroma, basally immersed in the uppermost layer of the stroma, dark olive green to black when dry, black in water; in 3% KOH with a reddish tinge, reversible after addition of LA, no pigment dissolved. Ostiolar dots (24–)31–42(–47) μm diam (*n* = 33), umbilicate, black.

Subperithecial and basal tissue of the stroma mostly consisting of a *t. angularis* of thin-walled, hyaline cells (6–)7.5–15(–18.5) × (3.5–)5–8.5(–11) μm (*n* = 30), becoming hyphal adjacent to the host tissue; stroma tissue without colour change in KOH or LA. Perithecia (205–)216–271(−277) μm high, (153–)171–234(–250) μm wide (*n* = 12), globose to subglobose, partially immersed in stroma, apical parts exposed. Ostioles periphysate, periphyses 12–34 μm long, 1.2–2 μm wide (*n* = 10). Peridium 35–90 μm thick, consisting of three layers: a 6–24-μm thick inner layer of hyaline to subhyaline, thick-walled (outermost) to thin-walled (innermost) elongate cells (6–)8–15(–19) × (1–)2–4(–5) μm (*n* = 50); a 13–24-μm-thick medium layer of dark olive green, thick-walled, elongate cells (6–)8–15(–18.5) × (4–)5–8(–11) μm (*n* = 30) turning red brown in KOH and olivaceous to umber brown in LA; and a 16–49-μm-thick outer layer of subhyaline to light olive green, thick-walled, elongate to isodiametric cells (5.5–)6.5–10.5(–12.5) × (3–)3.5–5.5(–7) μm (*n* = 30); surface sometimes covered by a thin outer layer of collapsed cells and amorphous material. Asci oblong, narrowly clavate or fusoid, lacking a differentiated apical apparatus, upper part with eight uniseriate ascospores (45–)53–66(–72) × (8–)9–10.5(–11) μm (*n* = 27), lower part stipe-like, ca. 8–20 μm long. Ascospores (8.5–)9.5–11(–12.5) × (4.0–)4.5–5(–5.5) μm, l/w (1.6–)1.9–2.4(–2.9) (*n* = 130), ellipsoid, oblong or fusoid, hyaline, with a median or slightly eccentric septum, straight, symmetric or slightly curved, slightly constricted at the septum, with broadly rounded ends, distinctly verruculose in water and LA, smooth in 3% KOH, with one large guttule per cell. Asexual morph on natural substrate not seen.

Cultures and asexual morph: colonies slow-growing, reaching 29 mm diam in 10 days on CMD; on MEA compact, flat, with white surface and yellowish reverse, after 1 month irregularly lobate, greyish brown in the centre, ochraceous with whitish patches at the margin; on CMD cottony, white, with abundant surface mycelium of hyphae commonly aggregated to hyphal strands, reverse yellowish. Conidiophores consisting of intercalary phialides with short lateral conidiiferous pegs (0.7–)0.8–3.0(–4.3) × (0.9–)1.1–1.6(–1.8) μm (*n* = 22), and terminally and laterally formed phialides. Phialides abundant on aerial mycelium, lageniform to cylindrical, (3–)7–15.5(–22) × (1.2–)1.6–2.3(–2.5) μm (*n* = 40). Conidia (3.5–)4.5–5.5(–6.5) × (1.3–)1.5–2.0(–2.5) μm, l/w (2.0–)2.6–3.3(–3.7) (*n* = 100), unicellular, allantoid, hyaline, smooth, commonly with a guttule at or towards one or both ends; after few days swelling to irregular shapes and up to ca. 9 × 3.5 μm. Blastoconidia formed on CMD in masses in the colony centre a few days after inoculation, hyaline, first ellipsoid to subglobose, globose and thick-walled with age, (2.5–)3–4.5(–5.5) μm diam (*n* = 120).

Distribution: Only known from a single collection in Portugal

Host: On dead corticated branches of *Quercus ilex*; probably saprobic

Holotype: Portugal, Parque Natural de Sintra-Cascais, S Monserrate, on *Quercus ilex*, 18 Feb. 2017, H. Voglmayr (WU 39972); ex-holotype culture NQI = CBS 144627; ex-holotype sequences MH562717 (ITS-LSU rDNA), MH562715 (*rpb1*), MH577042 (*rpb2*), MH562714 (*tef1*)

### Discussion

**Fig. 5:**
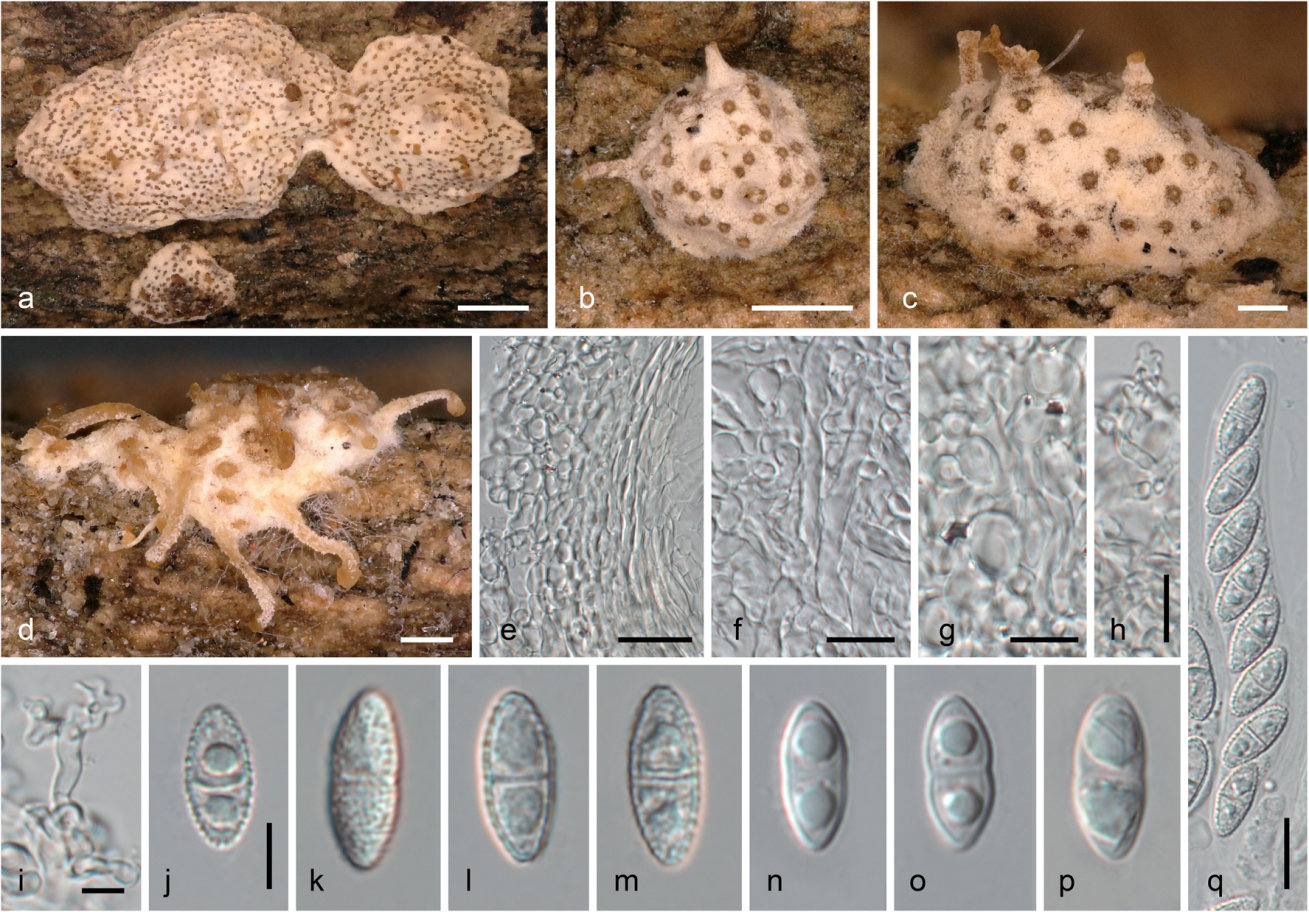
*Stilbocrea macrostoma* (**a**–**d**, **f**, **h**–**j**, **n**, **o** WU 32032; **e**, **g**, **k**–**m**, **p**, **q** WU 26101). **a**–**d** Stromata (**b**–**d** showing stilbella-like asexual morph). **e** Peridium in vertical section. **f**–**h** Stroma tissue in vertical section (**f** below perithecia; **g** stroma basis; **h** stroma surface). **i** Irregularly branched hyphae from stroma surface. **j–p** Ascospores (**j–m** in water; note verruculose and smooth ascospore walls in water and KOH, respectively). **q** ascus with ascospores (in water). (e–q in 3% KOH except where noted). *Scale bars***a** 1 mm; **b** 500 μm; **c**, **d** 200 μm; **e** 20 μm; **f**–**h**, **q** 10 μm; **i**–**p** 5 μm

In the phylogenetic analyses (Figs. 1 and 2), the fungus described here was unexpectedly placed in Bionectriaceae. Dark stromata and/or ascomata are not typically seen in Hypocreales, although they are formed by numerous nectriaceous species such as *Nectria eustromatica* (Jaklitsch and Voglmayr 2011b) or *Thyronectria obscura* (Jaklitsch and Voglmayr 2014). The species also shows a KOH-positive reaction of the ascomatal wall which is commonly seen in Nectriaceae (Rossman et al. 1999), but phylogenetic analyses of LSU sequences clearly placed the new fungus within Bionectriaceae, in a clade containing three accessions identified as *Stilbocrea macrostoma* (Fig. 1). Based on morphological distinctness, we consider the specimen from Portugal to represent a new species, described here as *S. walteri*. It differs substantially from *S. macrostoma*, and all putative synonyms listed in Seifert (1985) and Rossman et al. (1999), in its dark olive green to black perithecia, KOH and LA-positive reactions, compact stromata and a lack of a stilbella-like asexual morph. *Stilbocrea walteri* also contains much fewer perithecia which are apically free and only basally immersed in the stroma, whereas *S. macrostoma* contains numerous, up to several hundred ascomata almost entirely immersed in the stroma, resulting in a hypocrea-like appearance (Seifert 1985). Also, the stroma texture differs between the two species (a *textura angularis-globulosa* of thick-walled cells in *S. walteri*; a hyphal *textura intricata* with a surface layer of irregularly branched hyphae (cf. Figs. 2q and 4f–i; Seifert 1985, Rossman et al. 1999) in *S. macrostoma*). In addition, *S. macrostoma* is primarily a tropical to subtropical species, which to our knowledge has not been recorded from Europe. Notably, there are also a few characters of *Stilbocrea walteri* shared with *S. macrostoma*, like ascospores of similar size with a verruculose ornamentation disappearing in KOH (see Figs. 4e–v and 5j–q). Due to these marked discrepancies which could cast doubts on the reliability of the DNA sequences, DNA extraction was repeated directly from stromata, which revealed identical ITS-LSU sequences from stromata and culture, confirming that the sequences originate from the target fungus.

Our analyses (Figs. 1 and 2) may suggest that morphology of the sexual morph is not a good character for classification within Bionectriaceae and Nectriaceae. Asexual morphs, however, are not superior in this regard, as e.g. synnematous, stilbella-like asexual morphs also occur in the Nectriaceae, e.g. in *Nectria pseudotrichia* (Hirooka et al. 2012), and acremonium-like forms also in several other unrelated families of the Sordariomycetes (see, e.g. Summerbell et al. 2011). Also, the simple phialidic asexual morph of *S. walteri* observed in pure culture does not provide much phylogenetic information, as similar asexual morphs occur in various hypocrealean lineages.

Except for the commonly sequenced LSU, very few additional sequence data are available for most genera of Bionectriaceae. Apart from the well-studied genera *Geosmithia* and *Clonostachys*, even the ITS rDNA is lacking for many taxa. From the four species currently accepted in *Stilbocrea* (Rossman et al. 1999, de Beer et al. 2013), sequence data are available only for the generic type, *Stilbocrea macrostoma.* However, all three LSU sequences labelled as *S. macrostoma* differ substantially, and the two accessions from Sri Lanka and New Zealand form a highly supported subclade with the morphologically deviating *S. walteri* (Fig. 1), which is also seen in the analyses of the protein-coding genes (Fig. 2). Remarkably, this clade also contains two LSU sequences of endophyte isolates from the tropical marine seagrasses *Enhalus acoroides* (Sakayaroj et al. 2010) and *Thalassia hemprichii* (Supaphon et al. 2017), but unfortunately, no morphological data are available for them. In the LSU tree, the newly sequenced Panamese accession of *S. macrostoma* occupies a basal position in the poorly supported *Stilbocrea* clade (Fig. 1), but it is placed outside the *Stilbocrea* clade in the *rpb1* and *rpb2* trees (Fig. 2a, b), indicating that these accessions represent distinct species which may even not be congeneric. These results, together with the poor backbone support in the phylogenetic analyses (Figs. 1 and 2), suggest that a single gene alone is insufficient to provide a sound basis for defining phylogenetic generic concepts within the Bionectriaceae. A wide pantropical to warm-temperate distribution of *S. macrostoma* has been derived in the premolecular era (Seifert 1985), but if all sequences are correct in terms of generation from morphologically identical fungal material, then *S. macrostoma* will most probably be split into several species in future. Several taxa described from the Old and New World and synonymised with *S. macrostoma* based on morphology (Seifert 1985, Rossman et al. 1999) will then need to be re-considered and re-examined in detail. Remarkably, in their description of *S. macrostoma*, Seifert (1985) and Rossman et al. (1999) mentioned ascomata occasionally becoming red-brown to dark olive green with age; however, we have not seen any dark green colour in our material investigated. Much more sampling and generation of molecular data including protein-coding phylogenetic markers of Bionectriaceae are necessary to reveal a clearer picture of phylogenetic relationships within the family and to achieve a robust species classification and delimitation.
